# Integration of Visual and Joint Information to Enable Linear Reaching Motions

**DOI:** 10.1038/srep40869

**Published:** 2017-01-19

**Authors:** Henry Eberle, Slawomir J. Nasuto, Yoshikatsu Hayashi

**Affiliations:** 1 Brain Embodiment Lab, Department of Biomedical Engineering, School of Biological Sciences, University of Reading, Reading, RG6 6AH, United Kingdom

## Abstract

A new dynamics-driven control law was developed for a robot arm, based on the feedback control law which uses the linear transformation directly from work space to joint space. This was validated using a simulation of a two-joint planar robot arm and an optimisation algorithm was used to find the optimum matrix to generate straight trajectories of the end-effector in the work space. We found that this linear matrix can be decomposed into the rotation matrix representing the orientation of the goal direction and the joint relation matrix (***M***_*JRM*_) representing the joint response to errors in the Cartesian work space. The decomposition of the linear matrix indicates the separation of path planning in terms of the direction of the reaching motion and the synergies of joint coordination. Once the ***M***_*JRM*_ is numerically obtained, the feedfoward planning of reaching direction allows us to provide asymptotically stable, linear trajectories in the entire work space through rotational transformation, completely avoiding the use of inverse kinematics. Our dynamics-driven control law suggests an interesting framework for interpreting human reaching motion control alternative to the dominant inverse method based explanations, avoiding expensive computation of the inverse kinematics and the point-to-point control along the desired trajectories.

Path planning for reaching tasks is a fundamental problem both in robotics^1^ and behavioural science^2^. While many control schemes can guarantee convergence of an end effector upon a target, controlling the path it takes is much more complex. This is because traditionally, once a target has been defined in the robot’s work space using its sensors, the necessary final pose to intercept the object must be calculated using some form of inverse kinematics: the explicit transformation of work space coordinates into joint angles. However, inverse kinematics are both computationally intensive and, more importantly, very sensitive to error. Small errors in the kinematic model used can translate into large deviations from the desired pose and unstable motion. Furthermore, if a robot is commanded to simply minimise the difference between its current joint state and a calculated final pose, it will execute a movement that is linear in terms of its joint space, but strongly curved in the work space in which it actually operates. While the end effector still converges upon the target, the trajectory is ultimately not determined in visual coordinates, making it difficult to navigate the external environment. Ideally, the end effector should execute a direct, straight path in the work space in which the target is defined. In order to produce a straight trajectory, most robot control systems must create an explicit path to the target. This requires a complete specification of the desired motion^3^ and the leading methods all require a detailed specification of the robot’s kinematics and/or dynamics^4^.

Human reaching motions have characteristic properties that distinguish them from those of robots, the most visible being that they are near-straight in the work space^5^. Human hand movements also have a distinctive ‘bell-shaped’ velocity profile that can be effectively described by the minimum jerk model. There are many theories of how this control is achieved, several of which rely on the existence of paired forward and inverse models used to calculate appropriate torques for any movement^6^. One model that does not rely on this assumption is the equilibrium point (EP) hypothesis, which holds that human motions are governed by the human central nervous system (CNS) modulating the resting length of muscles without reference to kinematics^7^. The EP hypothesis frames movements as the convergence of a dynamical system upon a stable point, rather than the product of a kinematic or geometric calculation. Although there is disagreement on what reference frame or frames the (CNS) operates in when executing reaching movements, the motions themselves are strongly influenced by visual feedback. It has been demonstrated in experiments by Wolpert *et al*.^8^ and Flanagan and Rao^9^ that by distorting subject’s visual feedback on the position of their hand, the curvature of their reaching movements can be increased, implying that the CNS is attempting to achieve a straight path in terms of the available visual feedback.

Some have stated that inverse dynamic models are a necessity for the control of the human body, arguing that it is the only way to execute specific motions with such a complex system^10^,^11^. This paper comes from the viewpoint that computational complexity can be saved on the part of the controller by exploiting the dynamic properties of the system (a robot in this case).

Many researchers have utilised vision as a basis for controlling robots, either transforming visual data into feedback for a conventional robot controller (position-based visual servoing)^12^ or attempting to align the robot’s view with a specific scene corresponding to a work space target (image-based visual servoing)^13^. This often involves continuously recomputing the system Jacobian to obtain the correct joint velocities to reach the target, requiring detailed information on the robot’s kinematics. Where this is not available, such as when a robot is physically modified, the central assumptions of kinematics-based control break down, and robust control can no longer be ensured.

Our study considered whether it is possible to cause an arm to generate a straight path with its end effector by correcting visual path deviations as they arise during movement. This does not require a precise kinematic model and is thus much less sensitive to erroneous or incomplete data on the robot’s link lengths and joint states. This method relies on the robot’s nature as a dynamic system moving in the work space, as opposed to being derived from the system’s statics, like the Jacobian transpose method. We thus also aim to demonstrate that this method is energy efficient compared to the Jacobian transpose method, based on this distinction.

It has been established that a control law based on a linear transformation of visual information can enable a robot end effector to converge on a target within a specific area of the work space^14^. It has also been shown that in a humanoid robot, the relationship between the binocular coordinates (viewing directions and vergence angle) of its end effector and the joint space coordinates of its arm is well-approximated by a linear transformation where the arm is positioned to occupy the front visual field of the robot^15^. However in these cases the linear transformation has been treated as an approximation of the kinematics, rather than being examined in terms of the dynamics.

In this paper, a control law based on a static linear transformation matrix ***M*** is used to complete the sensory-motor loop of a simulated robot arm, with the aim of creating straight movements in the end effector. The matrix ***M*** is reminiscent of the concept of a ‘body schema’, a dynamic representation of the body’s behaviour. In humans this is the element of body representation that allows an understanding of how the body’s elements will coordinate^16^,^17^. In the simulated robot, ***M*** encodes a dynamic relationship between the arm’s components that results in a stereotyped movement. Because ***M*** defines how the dynamics of the arm’s joints interact, rather than acting as a computational model of the dynamics themselves, it is much simpler than the kinematic and dynamic models typically employed in the field of robotics.

## Results

### Proposed Control Law

**Figure 1:**
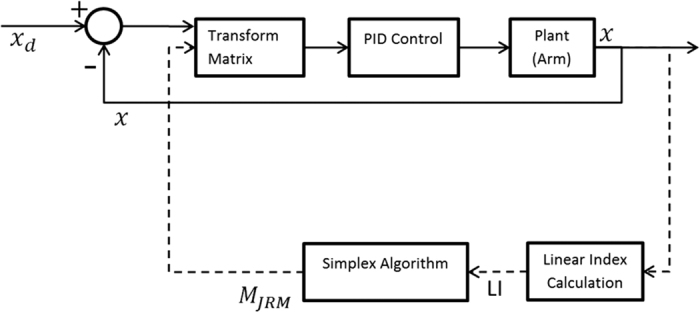
Block diagram of the robot control loop. The transform matrix is updated on a slow timescale in response to the performance of the control (the LI of its movements).

The control law used in this study aims to coordinate the dynamics of the robot’s individual joints such that its end effector moves in a straight line. To this end, a linear transformation matrix is applied to visual feedback to constrain the joint’s motion with respect to the work space, as can be seen in the system block diagram in Fig. 1. The proposed control law can be seen below:


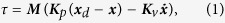


where ***M*** is a static transformation matrix applied to the visual information to alter the arm’s trajectory behaviour. ***τ*** is the vector of joint torques and ***x*** is the work space position of the end effector. ***x***_*d*_ is the target in the work space. ***K***_*p*_ is a proportional gain matrix for the work space error, while ***K***_*v*_ is a velocity gain matrix.

**Figure 2:**
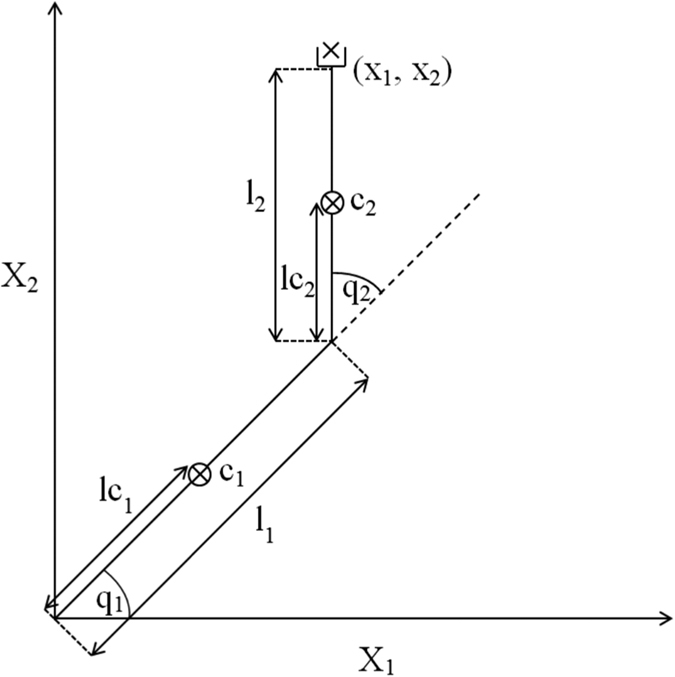
Image of the arm model: The location of the arm’s ‘hand’, ***x*** = [*x*_1_, *x*_2_], is calculated from the arm’s origin at its first joint. The vector ***q*** = [*q*_1_, *q*_2_] represents the rotation of the arm’s two joints, while *l*_1_ and *l*_2_ are the link lengths. The lengths of the sections connecting the links’ centres of mass (*c*_1_, *c*_2_) to the joints are represented by *l*_*c*1_ and *l*_*c*2_.

**Figure 3:**
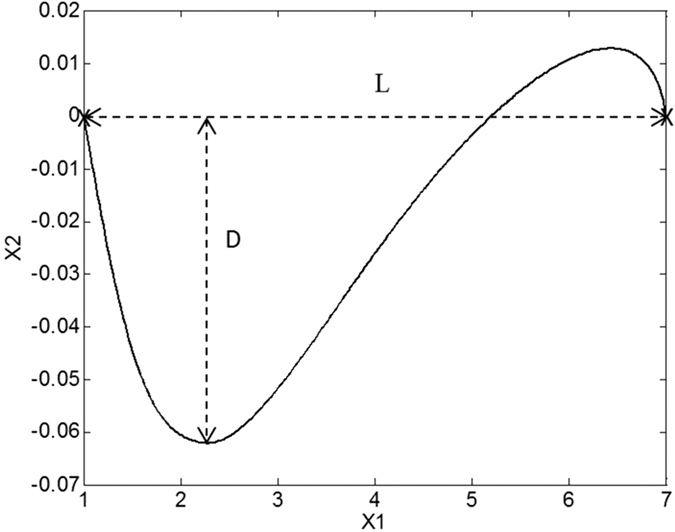
This figure shows the elements of the Linear Index (LI), which is a measure of the straightness of a path in Cartesian space. For this study *LI* = *D*/*L* Where *L* is the absolute distance between the starting and end positions of the robot’s end effector. *D* is the largest perpendicular deviation from the straight line vector that connects the start and end positions. In the line illustrated here, *L* = 6 and *D* ≈ 0.06, so the LI is 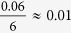.

Due to the two-dimensional nature of the model’s work space and joint space (illustrated in Fig. 2), ***M*** becomes a 2 × 2 matrix composed of four parameters that must be optimised to produce direct, straight line trajectories. We used a simplex optimisation algorithm to optimise ***M*** automatically to produce desirable motions, defined as those with a low Linear Index (LI). The LI (see Fig. 3), as utilised by Desmurget *et al*.^18^ is calculated by finding the greatest distance between the path taken by the end effector and the straight path connecting its starting and end points, as measured perpendicularly from the straight path:


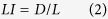


where *L* is the absolute distance between the starting and end positions of the robot’s end effector. *D* is the largest perpendicular deviation from the straight line vector that connects the start and end positions. The mass of the links was set at 1 arbitrary unit each, and their lengths at 4 within the simulation to obtain the results presented here, although the behaviour at larger masses and longer lengths is not qualitatively different.

### Properties of Simplex-Optimised Transformation Matrix

In order to eliminate the confounding effects of very large or small effective gains, each matrix produced by the optimisation algorithm was normalised by dividing it by its largest element. The optimised matrix was dependent on the orientation of the straight path from start point to target (*θ*), and appears to be the product of a 2D rotation matrix ***T***(−*θ*) and a constant matrix. At the angular origin of 0 radians on the X axis of the workspace the optimal matrix is 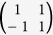, implying that this is the constant component, given this specific robot structure.

**Figure 4:**
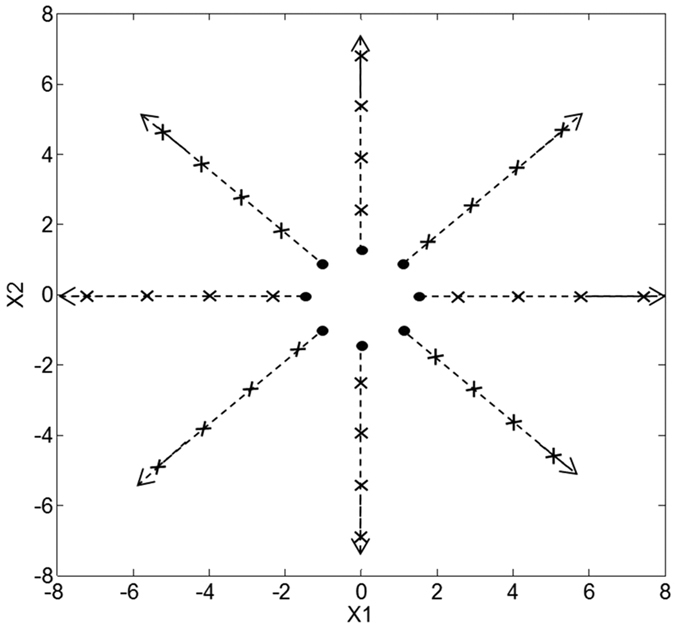
Radial motions are simulated with starting positions (shown as dots) initialised on a circle surrounding the origin, and targets (shown as crosses) placed at fixed distances up to the limit of the arm’s reach. Each start point/target pair represents a different orientation of movement (*θ*). The LIs of the resulting trajectories are then calculated and drawn onto the target point of each corresponding movement, producing a heatmap.

In order to validate that this was an invariant, ‘base’ component of ***M***, the procedure shown in Fig. 4 was used, where targets are sampled at evenly spaced polar coordinates and reaching motions to the targets are then simulated. By mapping the LIs of the resulting trajectories to their intended targets in the work space as a heat map, it is possible to see how ***M***’s effectiveness varies with the angle of motion. LI also acts as a proxy for the stability of the motion, as large values correspond with trajectories that oscillate uncontrollably rather than converging on the target.

**Figure 5:**
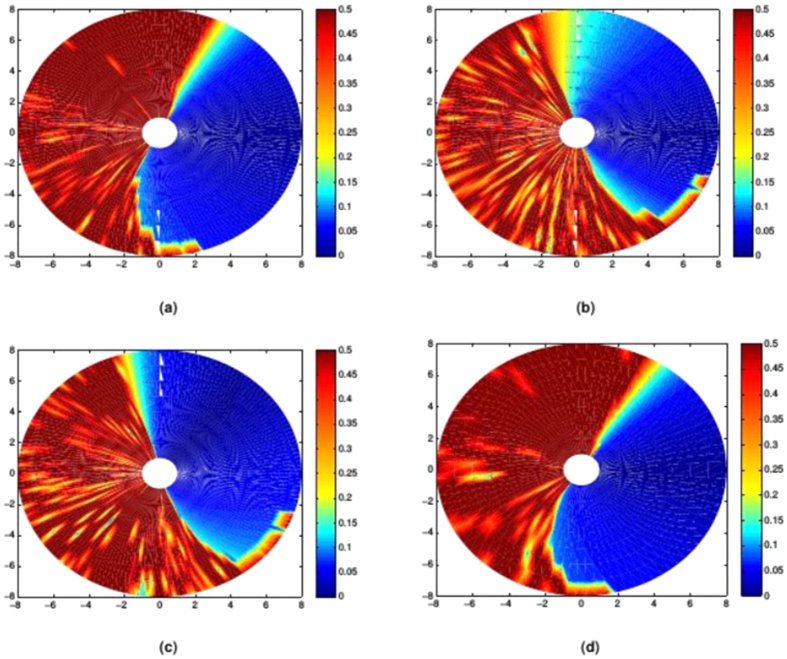
Comparison of four matrices optimised for movements with an orientation of 0 radians. The LI of outward reaching movements is visible as a heat map in the arm’s workspace, with the LI values mapped to the target of each movement. LI is saturated at higher values as these correspond to unstable trajectories where the measure becomes unreliable. (**a**) 
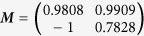
, arrived at using simplex optimisation. (**b**) 
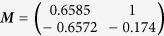
, arrived at using simplex optimisation. (**c**) 
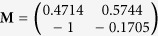
, arrived at using simplex optimisation. (**d**) 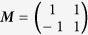, the hypothesised optimum value for movements with an orientation of 0 radians.

**Figure 6:**
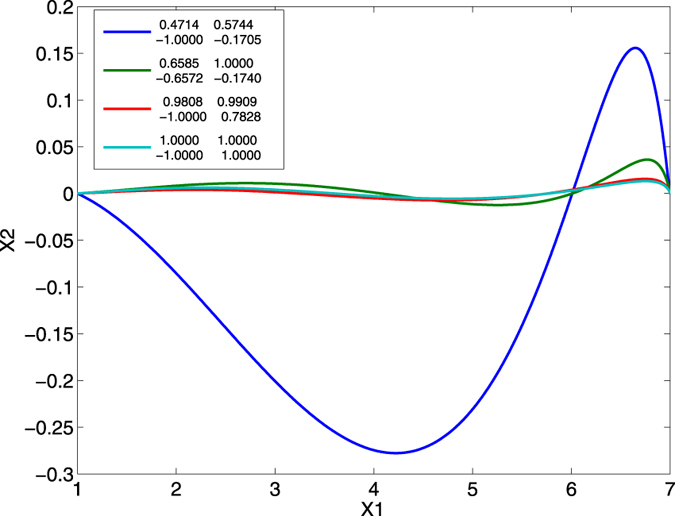
Paths taken by the end effector between the points (1, 0) and (7, 0), where M is set to a number of values. The first three matrices are the result of simplex optimisation, with each normalised by its largest value. The final matrix is the normalised 2D rotation matrix for −*π*/4 radians.

In Fig. 5 it can be seen that while each of the simplex-optimised matrices is associated with linear motions at 0 radians (example trajectories for these *M*_*JRM*_ values can be seen in Fig. 6), 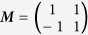. The closer the value of ***M*** to 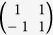 had the widest stable region and lowest minimum LI (the matrices have been normalised with respect to their largest element to discount the effect of a higher control gain). The considerations above lead to the hypothesis that the linear behaviour was not purely the property of a rotational transformation, and that the optimum ***M*** for a given radial motion could be factored into a rotational transformation and an invariant underlying matrix. These were denoted as a constant joint relationship matrix (***M***_*JRM*_), encoding the relationships between the motion of each joint in the arm and a 2D rotation matrix (***T***(−*θ*)) that aligns the coordinates of the target radial trajectory with the *X*_1_ axis of the work space:


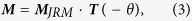


Thus, for the optimum ***M*** for any given orientation of movement, it was possible to post-multiply the final value by ***T***(−*θ*) to find if the value of ***M***_*JRM*_ was invariant.

### Properties of Analytically-Derived Transformation Matrix

In order to confirm that ***M*** could be decomposed into the components ***M***_*JRM*_ and ***T***(−*θ*), the path generation procedure in Fig. 4 was again used. The value of ***M***_*JRM*_ was set to 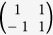, corresponding to the optimal value at 0 degrees and this was post-multiplied by a rotation matrix ***T**(θ*). If our hypothesis was correct, the combination of these two matrices would be the optimum ***M*** matrix for movements with the radial coordinate *θ*.

**Figure 7:**
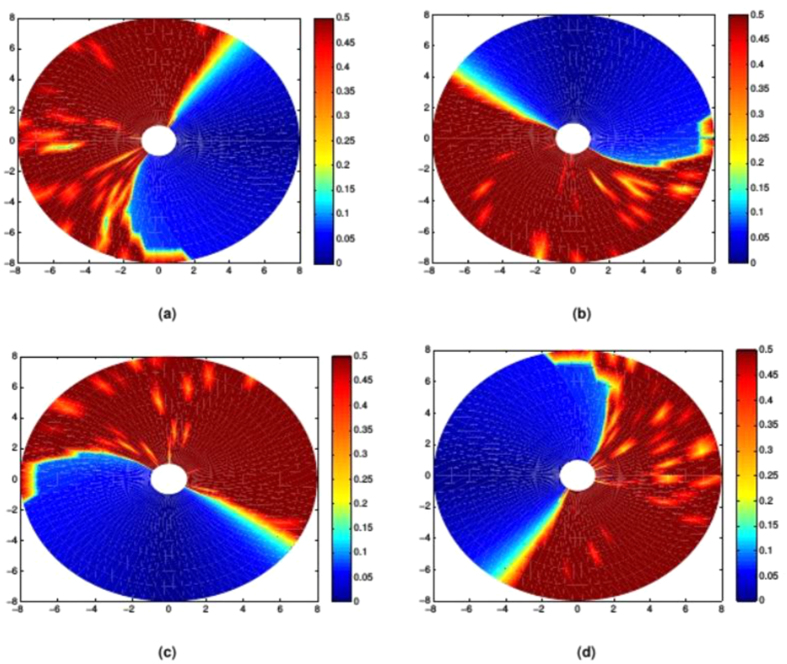
LI values for different *M* matrices where the *M*_*JRM*_ is kept constant at 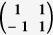 and the angle of *T*(−*θ*) varies by *π*/2 radians, 
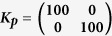
, 
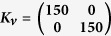
, *k* = 1, link lengths = [4, 4]. (**a**) *θ* = 0, 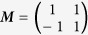. (**b**) *θ* = *π*/2, 
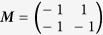
. (**c**) *θ* = 3*π*/2, 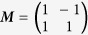. (**d**) *θ* = *π*, 
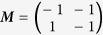
.

The test results are shown in Fig. 7, where it can be seen that there is a region of very low LI values (representing straight movements) occupying over a quarter of the work space. It can also be seen that multiplying the ***M***_*JRM*_ by a rotation matrix rotates this region by the specified number of radians in the opposite direction, as hypothesised.

Figure 7 shows LI values up to 0.5, as higher values represent unstable trajectories that have not converged on the target, making the calculation of LI unreliable. The transition between the low and high-LI areas is very abrupt, with a few degrees of moderately high LI leading directly into instability.

**Figure 8:**
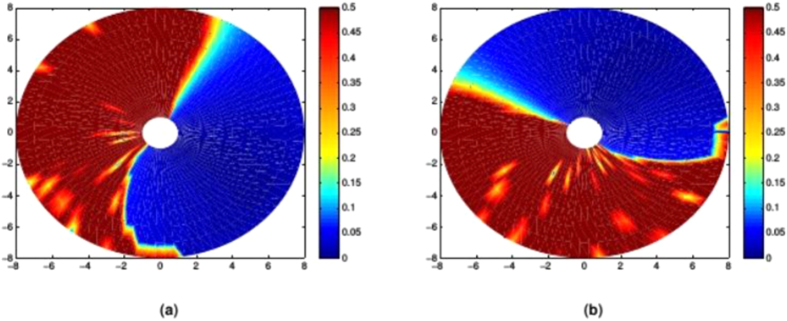
LI values produced where both visual and kinematic feedback is used. The ***M***_*JRM*_ remains constant at 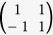 and the angle of ***T***(−*θ*) varies by *π*/2 radians, 
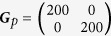
, 
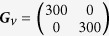
, *k* = 0.5, link lengths = [4, 4]. (**a**) *θ* = 0, 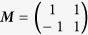. (**b**) *θ* = *π*/2, 
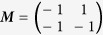
.

In order to see whether the stability of the control law would be improved or worsened by the inclusion of joint space feedback, Eq. 1 was altered to include joint space feedback using a target calculated with the arm’s inverse kinematics:


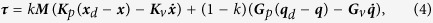


where ***q*** is the vector of joint rotations and ***q***_*d*_ is the joint space target calculated using the arm’s inverse kinematics (***q***_*d*_ = *f*^−1^(***x***_*d*_)). The variable *k* is a dimensionless scalar in the range 0 ≤ *k* ≤ 1 controlling the proportion of kinematic feedback used by the control law. The effect of including kinematic feedback can be seen by comparing Fig. 8 with Fig. 7, which shows that including kinematic feedback does not qualitatively affect the results, but does slightly expand the area in which the arm is stable. Adding the joint space feedback thus appears to have a stabilising influence, but only when sufficiently near the stable region for the work space terms.

### Mechanism of Joint Relationship Matrix

The fact that the control law defined in Eq. 1 produces near-straight trajectories (with the optimal ***M***_*JRM*_) can be understood in terms of the arm’s geometry and dynamics, instead of a coordinate transformation. Any movement of a revolute joint causes a curve in the trajectory of the end effector, deviating from the desired straight path. The ***JRM*** connects the torque at the joints to the motion of the end effector such that any deviation caused by one joint is compensated by the other. In this way, the motions of the two joints coordinate and the end effector remains on a straight trajectory.

**Figure 9:**
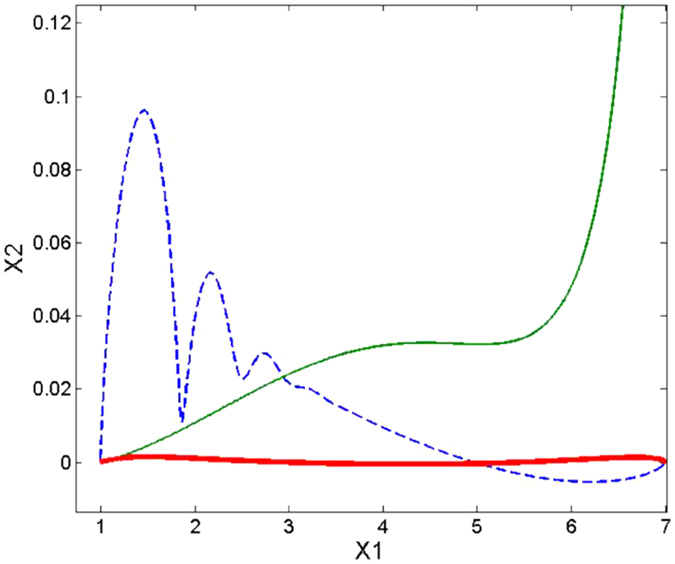
Instantaneous movement of a 2-joint planar arm. ***q*** = [*q*_1_, *q*_2_] represents the angles of the arm’s two joints. *r*_1_ and *r*_2_ represent the radii of the sub-movements caused by joints 1 and 2, respectively while ***T*** = [*T*_1_, *T*_2_] represents the magnitudes of the resulting tangential displacements of the end effector. For this example the ***M***_*JRM*_ is set to the identity matrix ***I***.

**Figure 10:**
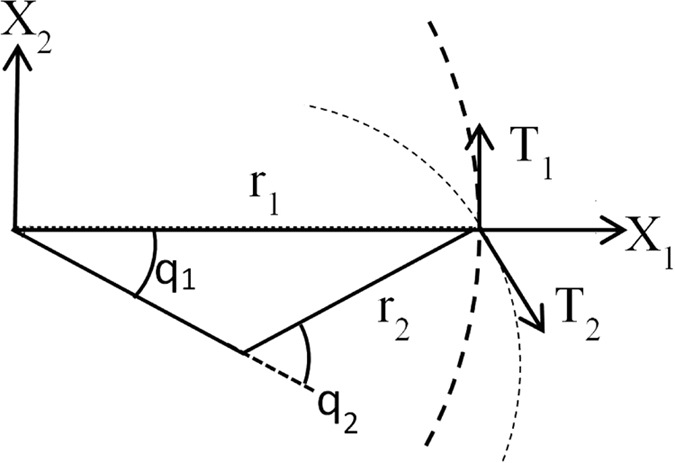
Trajectories between [1, 0] and [7, 0] where *M*_*JRM*_ equals 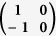 (solid line), 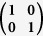 (dashed line) and 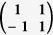 (thick line). 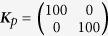
, 
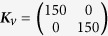
, *k* = 0, link lengths = [4, 4].

This section considers the simplest case of an arm with links of equal length where the end effector approaches a target on the positive *X*_1_ axis, with the second joint below it as pictured in Fig. 9. Because of this, there is no need to transform the coordinate system and ***M*** is equal to the ***M***_*JRM*_. The optimal ***M***_*JRM*_ matrix in this case has the form 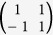. This form of matrix produces a motion of the end effector represented by the thick line in Fig. 10, indicating that the movements of the two joints are coordinated.

In order to understand the effect of the optimal ***M***_*JRM*_, it is useful to decompose the movement of the end effector into two superimposed movements, as illustrated in Fig. 9. The first is the rotation of the entire arm around the base joint of the first link, giving a circular trajectory of the end effector with radius equal to the distance between the base joint and the end effector (*r*_1_ in Fig. 9). The second movement is the rotation of the end effector around the second joint. This follows a circular path with a constant radius equal to the length of the second joint (*r*_2_ in Fig. 9), but also causes a change in the radius of the first sub-movement (*r*_1_). These two sub-movements are governed by the first and second rows of ***M***_*JRM*_, respectively.

Here we consider the tangential displacements *T*_1_ and *T*_2_ caused by the first and second sub-movements, respectively, in order to explain how the control law described by Eq. 1 maintains a straight path.

In order for the end effector to remain on a straight path, the vertical components of the tangents *T*_1_ and *T*_2_ must cancel, leaving a net horizontal movement of the end effector. Because the angle between *T*_1_ and *T*_2_ changes as the arm extends, in order to follow the *X*_1_ axis the velocity of joint 2 must change over time in order to maintain this balance. If it does not, the end effector will diverge from the straight path as seen in the solid line in Fig. 10. In practice, the control law described by Eq. 1 does not calculate the exact velocities needed, but relies on the fact that so long as the signs of the elements of ***M***_*JRM*_) are correct, the end effector will eventually be drawn onto the line connecting the arm origin to the target. As the first joint ‘extends’ the arm by accelerating in a positive direction, it causes the vertical component of *T*_1_ to grow, meaning that joint 2 must respond by accelerating in a negative direction to maintain balance. This is also why the transformation ***T***(−***θ***) is required in Eq. 3, as the necessary signs will differ with the orientation of the radial movement. The fact that all elements of the optimum ***M***_*JRM*_ have the same magnitude is a reflection of the fact that the links have the same length; the optimal ***M***_*JRM*_ gives the closest match in joint velocities over the course of the movement, requiring smaller reactive corrections.

**Figure 11:**
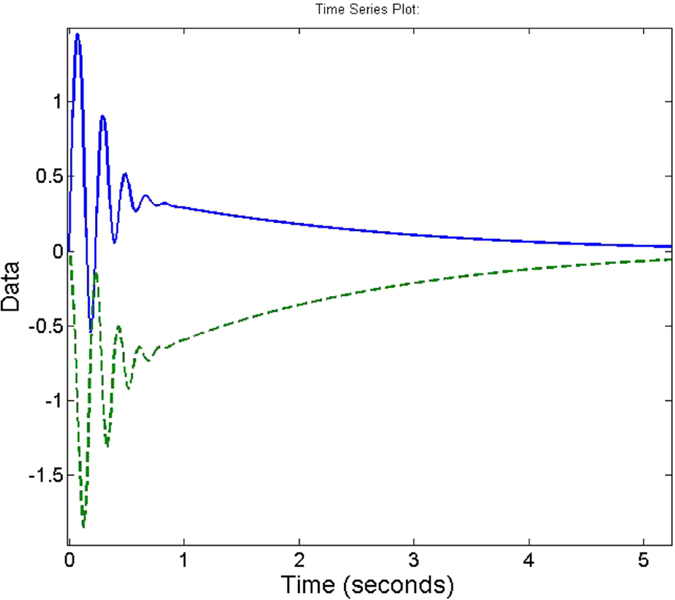
Velocities of joint *q*_1_ (solid line) and *q*_2_ (dashed line) where *M*_*JRM*_ equals 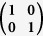.

The dashed line in Fig. 10 demonstrates how errors caused by one joint are corrected by the other when ***M***_*JRM*_ is set to the 2 × 2 identity matrix. In this case, the second joint reacts to the deviations caused by the first, but the speed of this correction is fundamentally limited by the response time of the joints (this can be seen in Fig. 11). In effect the problem of kinematic error has been replaced by one of temporal error; a failure of the joints to coordinate. The antidiagonal terms of the optimal ***M***_*JRM*_ counteract this effect with a basic form of forward modeling: feedback of opposing sign is fed into the two joints, relying on their similar dynamic responses to create the similar movements. The deviations that must be corrected are thus rendered smaller and the instability associated with delay in the sensory-motor loop is reduced.

### Energy Efficiency

The ***M***_*JRM*_ matrix utilised in this control law operates by extending the arm, while simultaneously correcting any deviations from a straight line. We investigated whether this corrective method could execute a straight path while using relatively little energy, as opposed to if the torques were set directly by kinematic calculation. To that end this section measures the sum of squared torque (proportional to energy used for a DC motor) used by the ***M***_*JRM*_ (set at 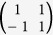) and the transpose of the arm Jacobian, when substituted into the control law (Eq. 1). Our paper does not distinguish between motor and braking torques because it is attempting to characterise an upper estimate in the absence of any mechanism for energy storage or restoration. It is intended to be a comparison between the velocity profiles produced by each control strategy, each of which requires the motors to exert a different amount of torque.

The Jacobian transpose is often used in closed loop control of robot manipulators as a means of transforming desired end effector forces into joint torques (***J***^*T*^***F*** = ***τ***). The justification for this use is based on the relationship between end effector forces and joint torques defined by the virtual work principle:


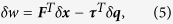


where *δw* is the change in the work performed by the arm, ***F*** is the vector of forces at the end effector, ***τ*** is the vector of joint torques, *δ**x*** is change in the Cartesian coordinates of the end effector and *δ**q*** is the change in joint coordinates. This states that end effector forces can be directly transformed into joint torques where the arm is in equilibrium.

**Figure 12:**
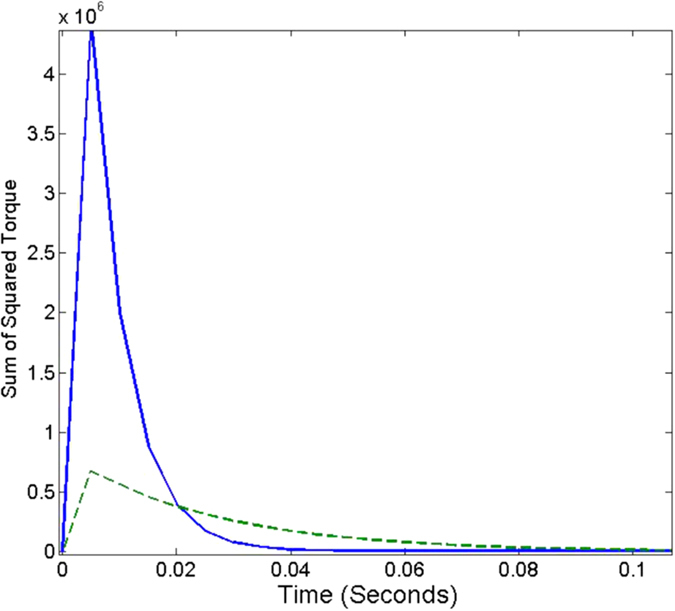
Comparison of torque applied during a point to point motion from [1, 0] to [7, 0] using the *M*_*JRM*_ 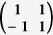 (dashed line) and the Jacobian transpose (solid line) as the transformation matrix.

**Figure 13:**
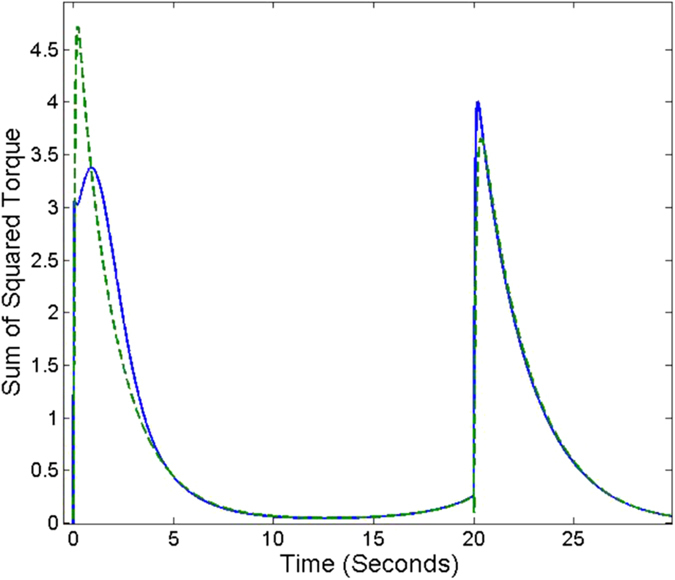
Comparison of torque applied tracking a point moving from [1, 0] to [7, 0] at a speed of 0.05 per second using the *M*_*JRM*_ 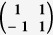 (dashed line) and the Jacobian transpose (solid line) as the transformation matrix.

As can be seen in Figs 12 and 13, respectively, the ***M***_*JRM*_ method is considerably more efficient than the Jacobian transpose at simple point-to-point movements, but the difference is much less marked where the arm is tracking a moving equilibrium point. However, while the Jacobian transpose uses more energy to track at lower control gains, the energy used by the ***M***_*JRM*_ method slightly decreases.

We postulate that this is because the Jacobian transpose’s use as a transformation comes from the arm’s statics, while the act of reaching requires the arm to perform work. This renders the direct relationship between forces and torques invalid and leaves the Jacobian transpose as an inappropriate transformation so long as the arm is out of equilibrium. In contrast, our method’s assumptions are not violated if the arm is in motion.

### Stability Analysis

Lyapunov’s direct method was employed to prove the stability of the control law described by Eq. 1 when applied to a simulated robot arm with dynamics described by Eq. 19. Firstly, a Lyapunov candidate was defined:


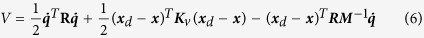


In order to prove *V* is positive-definite, we rewrite the expression in matrix form:


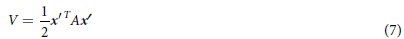


where:


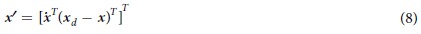


and


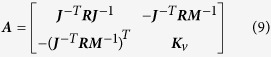


where ***J*** is the Jacobian 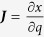. As ***A*** is a symmetric 2 × 2 block matrix; its Schur complement can be used to prove that it is positive definite. Thus ***A*** > 0 (and thus *V* > 0) iff:

1. ***K***_*v*_ > 0
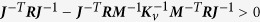

The first inequality is always true, as ***K***_*v*_ is a positive diagonal matrix. Simplifying, and using the knowledge that ***K***_*v*_ = *k*_*v*_***I***, the second inequality becomes:


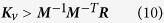


Thus, setting ***K***_*v*_ to a sufficiently large positive value will ensure that *V* is positive definite.

Differentiating *V* with respect to time gives:


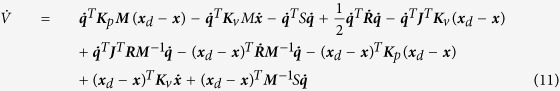


where ***R*** is the positive-definite, symmetric inertial matrix and ***S*** is a matrix of Coriolis forces. 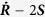 is a skew-symmetric matrix, which means that:


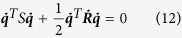


and thus in matrix form:


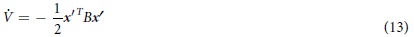


where:


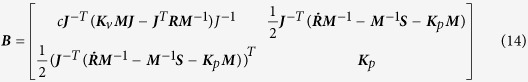


***B*** is not a symmetric matrix, but the Schur complement of its Hermitian part can be calculated yielding 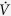 negative definite (and Eq. 1 asymptotically stable) iff:

1. ***K***_*p*_ > 0.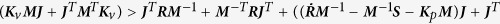 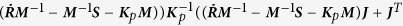 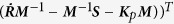.

The first inequality is true for all ***K***_*p*_ > 0 and thus applies to any diagonal gain matrix of the form ***K***_*v*_ = *k*_*v*_***I***. The second inequality holds if (***K***_*v*_***MJ*** + ***J***^*T*^***M***^*T*^***K***_*v*_) > 0, both ***K***_*v*_ and ***K***_*p*_ are sufficiently large and ***K***_*v*_ is sufficiently larger than ***K***_*p*_. In particular, since ***S*** and 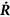 are unbounded in 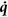, the control gains set an effective constraint on the arm velocity, in that the gains must be large enough to counteract the Coriolis and other forces that act on the moving arm. Beyond the geometric constraints related to the end effector’s location in the work space, these stability conditions are very similar to those of a traditional kinematics-based control law with a proportional-derivative control loop.

## Discussion

This study demonstrates that the otherwise complex task of planning a straight path for an end effector can be solved without explicitly defining the path itself. This is achieved by exploiting the dynamic relationship between an open chain robot’s components. The fact that the movement of any link affects all others further down the chain allows the implementation of a visual-motor loop that continuously corrects deviations from the desired path without relying on accurate kinematics, which are not available for all robotic systems.

This study demonstrates that the otherwise complex task of planning a straight path for an end effector can be solved without explicitly defining the path itself. This is achieved by exploiting the dynamic relationship between an open chain robot’s components. The fact that the movement of any link affects all others further down the chain allows the implementation of a visual-motor loop that continuously corrects deviations from the desired path without relying on accurate kinematics, which are not available for all robotic systems.

A consequence of this is that not all standard means of comparison can be used in this case, as the tests rely on assumptions that are not applicable to our study. Tracking tests, for example, cannot be properly applied because the control is optimised for a cost function calculated over an unconstrained trajectory as no true reference trajectory exists, only a start point and target.

The simplicity of the ***M***_*JRM*_ matrix and the wide area rendered stable by any given value also allow stable reaching motions to be achieved much more simply than in the classical robot model. The transformation matrix can easily be generalised over the entire work space by applying a rotational transformation. This transformed visual feedback can be linearly combined with conventional inverse-kinematics based error to add more stability, but if accurate encoder or visual sensors are available it is unlikely to be necessary.

Furthermore, the ***M***_*JRM*_ method exerts less torque (and thus uses less energy) to move when in a non-equilibrium state than the Jacobian transpose. This could render it useful in situations where sufficient control gain to rigidly track a moving equilibrium point is not possible.

In future work we hope to apply the same self-correcting behaviour to arbitrary motions within the work space, allowing true global stability to be achieved. The degree to which this method can be extended to systems with many degrees of freedom is a matter of continued investigation. There is preliminary evidence that redundant arms controlled using this method experience continual self-motion unless appropriate joint limits are imposed.

## Methods

### Numerical Simulation of a Planar Arm

The control scheme developed in this study was tested using a numerical simulation of a two-joint planar robot arm. This is the simplest form of an arm capable of executing the ‘reaching’ motions the study aims to optimise. This reduced to a minimum the number of parameters that had to optimised while retaining the classical robot arm form.

Figure 2 shows the arm model used in this study, along with its primary attributes. The model’s sensors can be thought of as accurate encoders at the joints and an undistorted camera overlooking the arm in a bird’s-eye view to detect the end-effector position. The block diagram of the overall system can be seen in Fig. 1.

The model was implemented in Simulink, using a third-order ordinary differential equation solver with a constant step size of 0.005 s. The primary components of the simulation are the arm’s dynamics and the control law described by Eq. 1. Due to their relative complexity these are both implemented as custom MATLAB functions embedded in Simulink blocks. The control law block produces a time series of torque values that are input into the dynamics block to calculate the arm’s joint acceleration over time. This is integrated twice and used to determine the arm’s joint state. The position of the arm’s end effector and joints is calculated using the forward kinematics of the arm and treated as the visual feedback.

The reaching target is simply modeled as a constant Cartesian coordinate vector.

### Dynamics

The links of the arm are modeled as thin, uniform rods with the moment of inertia *j* = *ml*^2^/3 (where *m* is the link mass and *l* is the link length) and their centre of mass halfway along their length. In the case we are modeling, the links have the same mass (*m*_1_ = *m*_2_) and equal length (*l*_1_ = *l*_2_). The arm is considered to be supported by a parallel plane, reducing the gravity term to 0. This restriction to a fixed plane replicates the constraints placed upon human subjects in many reaching experiments^8^,^9^.

The dynamics of the simulation are derived by solving the Lagrange equation for a driven robotic system:


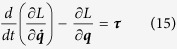


where ***τ*** is the vector of joint torques, ***q*** is the vector of joint angles and *L* = *V* − *U* where *V* and *U* are the kinetic and potential energy, respectively. Because no gravity is experienced by the arm, *U* is set to 0.


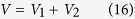



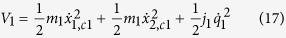



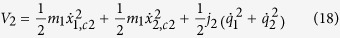


where *x*_1,*cn*_ and *x*_2,*cn*_ are the *x*_1_ and *x*_2_ coordinates of the nth joint’s centre of mass, respectively.

Solving Eq. 15 gives the final dynamics equation:


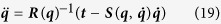


with the inertial matrix:


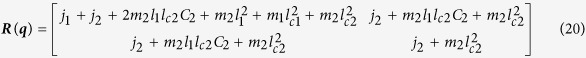


and a Coriolis force vector with the form below.


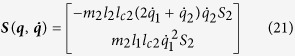


*C*_*n*_ = cos *q*_*n*_ and *S*_*n*_ = sin *q*_*n*_. Equation 19 is used to calculate the acceleration of the arm joints given the control torques. No friction is modelled at the joints (this does not weaken the stability conditions for control).

### Simplex Optimisation

In order to optimise the transformation matrix *M*_*JRM*_ we used the widely-known simplex algorithm defined by Nelder and Mead^19^. Each element of the simplex is a value for the transformation matrix *M* in Eq. 1 (each with 4 elements, giving a 5-point simplex). The error for each matrix was acquired by substituting it into Eq. 1 and simulating a point-to-point movement of the robot in which all other parameters were fixed, before calculating the LI (defined in Eq. 2). By using this algorithm to continuously generate new matrices, it was possible to find the optimal value of *M*_*JRM*_ for a specific start/endpoint pair. Unlike the original formulation of the simplex algorithm, the algorithm used here does not include the ‘contraction’ step that reduces the simplex’s deviation around the mean if the projected new value has a sufficiently high error. Not including this step improved the algorithm’s likelihood of finding a good solution in all the experiments performed.

The initial simplex to be optimised was generated by pseudo-randomly perturbing a ‘seed’ matrix. When performing individual ‘runs’ of experiments optimising for radial movements at a series of angles, this matrix was randomly chosen and kept constant throughout.

Because the end effector position is based on the angle of both joints, it was assumed that the components of ***M*** are inter-related, but without knowing this relationship, it was not possible to design a problem-specific algorithm. This limited the choice of algorithms to general optimisers. The simplex optimisation algorithm created by Nelder and Mead^19^ was a good fit in that it is often used to optimise sets of variables with unclear relationships. Additionally, algorithms of this form do not require the derivative of the problem, which could not practically be found due to the sheer number of simulations that would be required.

## Additional Information

**How to cite this article**: Eberle, H. *et al*. Integration of Visual and Joint Information to Enable Linear Reaching Motions. *Sci. Rep.* **7**, 40869; doi: 10.1038/srep40869 (2017).

**Publisher's note:** Springer Nature remains neutral with regard to jurisdictional claims in published maps and institutional affiliations.

## References

[b1] Hashimoto K. A review on vision-based control of robot manipulators. Advanced Robotics 17, 969–991 (2003).

[b2] Shadmehr R. The computational neurobiology of reaching and pointing: a foundation for motor learning (MIT press, 2005).

[b3] Macfarlane S. & Croft E. A. Jerk-bounded manipulator trajectory planning: design for real-time applications. Robotics and Automation, IEEE Transactions on 19, 42–52 (2003).

[b4] Nakanishi J., Cory R., Mistry M., Peters J. & Schaal S. Operational space control: A theoretical and empirical comparison. The International Journal of Robotics Research 27, 737–757 (2008).

[b5] Morasso P. Spatial control of arm movements. Experimental brain research 42, 223–227 (1981). 7262217 10.1007/BF00236911

[b6] Wolpert D. M. & Ghahramani Z. Computational principles of movement neuroscience. nature neuroscience 3, 1212–1217 (2000). 11127840 10.1038/81497

[b7] Feldman A. G. & Latash M. L. Testing hypotheses and the advancement of science: recent attempts to falsify the equilibrium point hypothesis. Experimental Brain Research 161, 91–103 (2005). 15490137 10.1007/s00221-004-2049-0

[b8] Wolpert D. M., Ghahramani Z. & Jordan M. I. Perceptual distortion contributes to the curvature of human reaching movements. Experimental brain research 98, 153–156 (1994). 8013583 10.1007/BF00229120

[b9] Flanagan J. R. & Rao A. K. Trajectory adaptation to a nonlinear visuomotor transformation: evidence of motion planning in visually perceived space. Journal of neurophysiology 74, 2174–2178 (1995). 8592205 10.1152/jn.1995.74.5.2174

[b10] Kawato M., Furukawa K. & Suzuki R. A hierarchical neural-network model for control and learning of voluntary movement. Biological cybernetics 57, 169–185 (1987). 3676355 10.1007/BF00364149

[b11] Wolpert D. M., Miall R. C. & Kawato M. Internal models in the cerebellum. Trends in cognitive sciences 2, 338–347 (1998). 21227230 10.1016/s1364-6613(98)01221-2

[b12] Wilson W. J., Williams Hulls C. & Bell G. S. Relative end-effector control using cartesian position based visual servoing. Robotics and Automation, IEEE Transactions on 12, 684–696 (1996).

[b13] Corke P. I. Visual control of robot manipulators-a review. Visual servoing 7, 1–31 (1993).

[b14] Nishida R. & Kawamura S. A new feedback robot control method based on position/image sensor integration. In Intelligent Robots and Systems (IROS), 2012 IEEE/RSJ International Conference on, 5012–5017 (IEEE, 2012).

[b15] Mitsuda T., Maru N., Fujikawa K. & Miyazaki F. Binocular visual servoing based on linear time-invariant mapping. Advanced robotics 11, 429–443 (1996).

[b16] Giummarra M. J., Gibson S. J., Georgiou-Karistianis N. & Bradshaw J. L. Central mechanisms in phantom limb perception: the past, present and future. Brain research reviews 54, 219–232 (2007). 17500095 10.1016/j.brainresrev.2007.01.009

[b17] Holmes N. P. & Spence C. The body schema and multisensory representation (s) of peripersonal space. Cognitive processing 5, 94–105 (2004). 16467906 10.1007/s10339-004-0013-3 PMC1350799

[b18] Desmurget M., Jordan M., Prablanc C., Jeannerod M. . Constrained and unconstrained movements involve different control strategies. Journal of Neurophysiology 77, 1644–1650 (1997). 9084629 10.1152/jn.1997.77.3.1644

[b19] Nelder J. A. & Mead R. A simplex method for function minimization. Computer journal 7, 308–313 (1965).

